# Empathic Neural Responses Predict Group Allegiance

**DOI:** 10.3389/fnhum.2018.00302

**Published:** 2018-07-31

**Authors:** Don A. Vaughn, Ricky R. Savjani, Mark S. Cohen, David M. Eagleman

**Affiliations:** ^1^Semel Institute for Neuroscience and Human Behavior, University of California, Los Angeles, Los Angeles, CA, United States; ^2^Department of Psychology, Santa Clara University, Santa Clara, CA, United States; ^3^Texas A&M University, College Station, TX, United States; ^4^Department of Psychiatry and Behavioral Sciences, Stanford University, Stanford, CA, United States

**Keywords:** empathy, pain, ingroup, machine learning, religion, social neuroscience, mind reading, affect

## Abstract

Watching another person in pain activates brain areas involved in the sensation of our own pain. Importantly, this neural mirroring is not constant; rather, it is modulated by our beliefs about their intentions, circumstances, and group allegiances. We investigated if the neural empathic response is modulated by minimally-differentiating information (e.g., a simple text label indicating another's religious belief), and if neural activity changes predict ingroups and outgroups across independent paradigms. We found that the empathic response was larger when participants viewed a painful event occurring to a hand labeled with their own religion (*ingroup*) than to a hand labeled with a different religion (*outgroup*). Counterintuitively, the magnitude of this bias correlated positively with the magnitude of participants' self-reported empathy. A multivariate classifier, using mean activity in empathy-related brain regions as features, discriminated *ingroup* from *outgroup* with 72% accuracy; the classifier's confidence correlated with belief certainty. This classifier generalized successfully to validation experiments in which the *ingroup* condition was based on an arbitrary group assignment. Empathy networks thus allow for the classification of long-held, newly-modified and arbitrarily-formed ingroups and outgroups. This is the first report of a single machine learning model on neural activation that generalizes to multiple representations of ingroup and outgroup. The current findings may prove useful as an objective diagnostic tool to measure the magnitude of one's group affiliations, and the effectiveness of interventions to reduce ingroup biases.

## Introduction

Neuroimaging reveals that watching another person in pain activates brain areas involved in the sensation of our own pain (Singer, 2004; Botvinick et al., 2005; Hein and Singer, 2008; Valeriani et al., 2008; Jacoby et al., 2016). Importantly, this neural mirroring is not constant; rather, it is modulated by our beliefs about their intentions, circumstances, and group allegiances. For example, there is a diminished response in this empathy network for pain if the observer believes the pain-recipient has acted unfairly in a simple economic exchange (Singer et al., 2006). A similar reduction occurs when the observer is told that the victim is receiving a large monetary compensation for undergoing the pain (Guo et al., 2011).

Modulation of empathy-associated activity occurs with group distinctions, as well. A larger activation for ingroups (vs. outgroups) has been demonstrated in the context of sports teams (Hein et al., 2010; Cikara et al., 2011), and racial identity (Xu et al., 2009; Azevedo et al., 2013; Contreras-Huerta et al., 2013). Generally, this ingroup bias translates into actions: neural activation in empathy-related areas predicts prosocial action (Hein et al., 2010; Christov-Moore and Iacoboni, 2016). Thus, understanding and quantifying these biases has important practical considerations, from jury decision-making to group profiling to genocides. However, it is unknown whether differences in low-level empathic biases are induced by ingroup/outgroup distinctions more generally, and how fluidly they can change.

In the current experiments, we sought to evaluate: (1) whether brain responses in empathy-associated areas differ between minimalistic representations of religious ingroups and religious outgroups, (2) whether the observed brain responses are related to self-reported empathy, (3) if multivariate brain responses reliably predict participants' ingroup and outgroup conditions, and (4) whether these differential empathic responses extend to loose and arbitrary ingroup and outgroup categories.

## Materials and methods

### Participants

We recruited 135 participants (29 ± 9 years, 63 males, 108 right-handed) with normal or corrected-to-normal vision. We used flyers posted around the greater Houston area (e.g., police stations, fire stations, and community centers) to recruit a wide range of participants. This recruiting approach successfully captured a diverse group with varied backgrounds. Participants were compensated for their time.

Data from 8 participants were excluded due to errors on MR image acquisition or reconstruction, and 22 participants were excluded from analysis due to excessive head motion (absolute mean displacement > 3.0 mm), leaving 105 participants in total for analysis. Of these 105 participants, 67 participants were used in Experiment 1, and a subset of 14 of those participants were used in Experiment 2. Separately, 14 participants were involved in Experiment 3. Importantly, 24 participants were involved in Experiment 1 or Experiment 2, but their data were not used in the ingroup/outgroup analyses, as they professed their religion to be agnostic. All 105 participants underwent the baseline block (see below) with neutrally-labeled hands, and their data were used in the functional localization of the empathy and relief networks. However, there was no overlap in participants between the three experiments; thus, the three ingroup/outgroup analyses were independent.

Participants were told they were being recruited for a study on the relationship between pain and memory. The study was classified as deceptive research since our true interest—understanding the neural empathic response—was not disclosed to participants. We conducted the collection of this data at Baylor College of Medicine (BCM) while authors DAV, RRS, and DME were (but no longer are) BCM employees. The study was approved by the BCM Institutional Review Board (IRB), as the protocol was deemed to be of no potential harm. Each and all subjects read, agreed to, and signed a written consent form, which was also reviewed and approved by the BCM IRB.

### Behavioral questionnaires

First, we asked participants to declare their religious belief as specifically as possible (including “agnostic” or “atheist”). The participants' self-reported religious affiliations were distributed as follows: 24 agnostics, 11 atheists, 49 Christians, 4 Hindus, 2 Jews, 1 Muslims, 0 Scientologists. Next, participants completed a brief survey that quantified empathy (Mehrabian, 1996)—Balanced Emotional Empathy Scale (BEES)—and degree of religious conviction. The religious conviction scale was adapted to map onto a portion of Richard Dawkins' 7 point scale, replacing “religion” by “religious belief” (Dawkins, 2008). A value of 1–4 on his scale corresponds to 0–3 on our scale; thus the 0 on our modified scale corresponds to complete uncertainty in a religious belief and 3 to complete certainty. A Christian who identifies culturally but not ideologically might respond with a 0, while a completely certain atheist would respond with a 3. Participants' mean response score on this metric was 2.85 ± 0.99.

### Stimuli

All stimuli were programmed in MATLAB (The Mathworks Inc., Natick, USA) with PsychToolbox (Brainard, 1997). Participants viewed the stimuli on a back-projected screen while lying supine in the scanner (see Supplementary Movie M1).

### Baseline block

**Figure 1:**
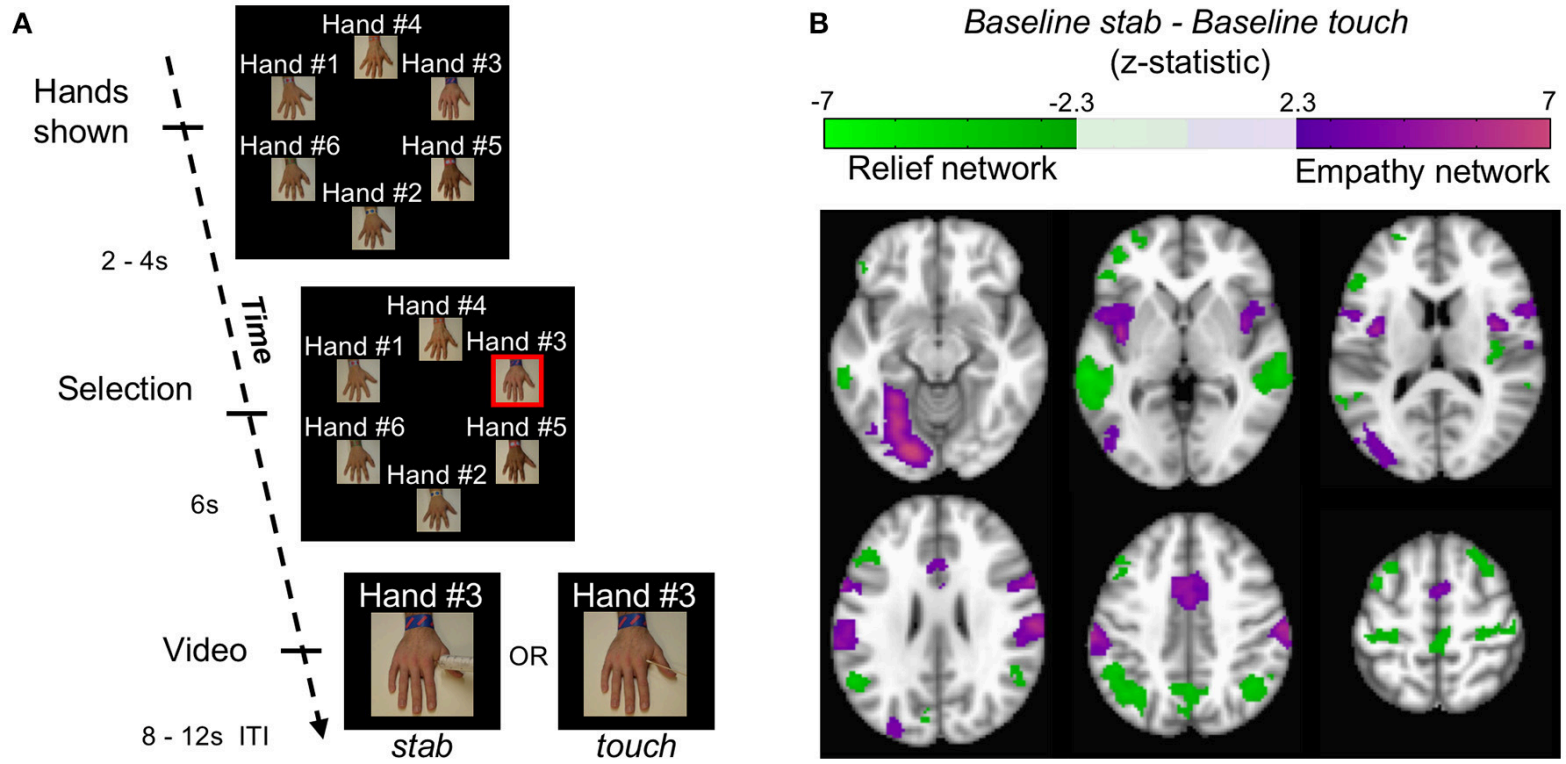
Localizing empathy. **(A)** In each trial, hands appeared with impartial text labels. The computer selected a hand, and that hand received either a stab or a touch. **(B)** Whole brain contrasts of *baseline stab*>*baseline touch* and *baseline touch*>*baseline stab* yielded 6 and 7 significant clusters, respectively (*p* < 0.05 FWE) shown here in MNI coordinates from −12 mm to 58 mm in 14 mm increments.

Using blood-oxygenation level dependent (BOLD) signal from functional magnetic resonance imaging (fMRI), we implemented a simple functional localization paradigm to identify regions involved in pain-related empathy. During each of 12 trials in this baseline block, a participant saw 6 hands appear on the screen (labeled neutrally as “Hand #1,” “Hand #2,” etc.). Each hand was similar in skin tone as well as apparent age and differentiated from others by an arbitrarily-assigned bracelet and text label that was intended to give each hand a unique identity. Two to four seconds later, one hand was selected randomly by the computer, indicated by the addition of a red border around the image. After 6 s, the selected image moved into the middle of the screen and became a 2.3 s video of that hand being stabbed with a needle (*baseline stab)*, or, alternatively, touched with a cotton swab (*baseline touch*) (Figure 1A, Figure S1, Supplementary Movie M1). During each trial the position of these hands on the screen was randomized. The text label remained with the hand to which it was assigned.

Because each participant saw multiple *stab* and *touch* trials in the course of an experiment, we filmed stabbing and touching events from six different angles to reduce desensitization. In most versions of the experiment, except as noted below, participants began by observing 6 *stab* trials and 6 *touch* trials; the contrast of these *baseline* conditions served as a functional localizer for us to define the *empathy* and *relief* networks operationally. Observations were separated by a blank screen of at least 8–12 s (the inter-trial interval). The display position of each hand and its associated text label was shuffled for each trial. Participants then were assigned to one of three experimental conditions, which were identical in structure, but different in the construction of *ingroup* and *outgroup* conditions.

## Experiment 1: ingroup vs. outgroup

**Figure 2:**
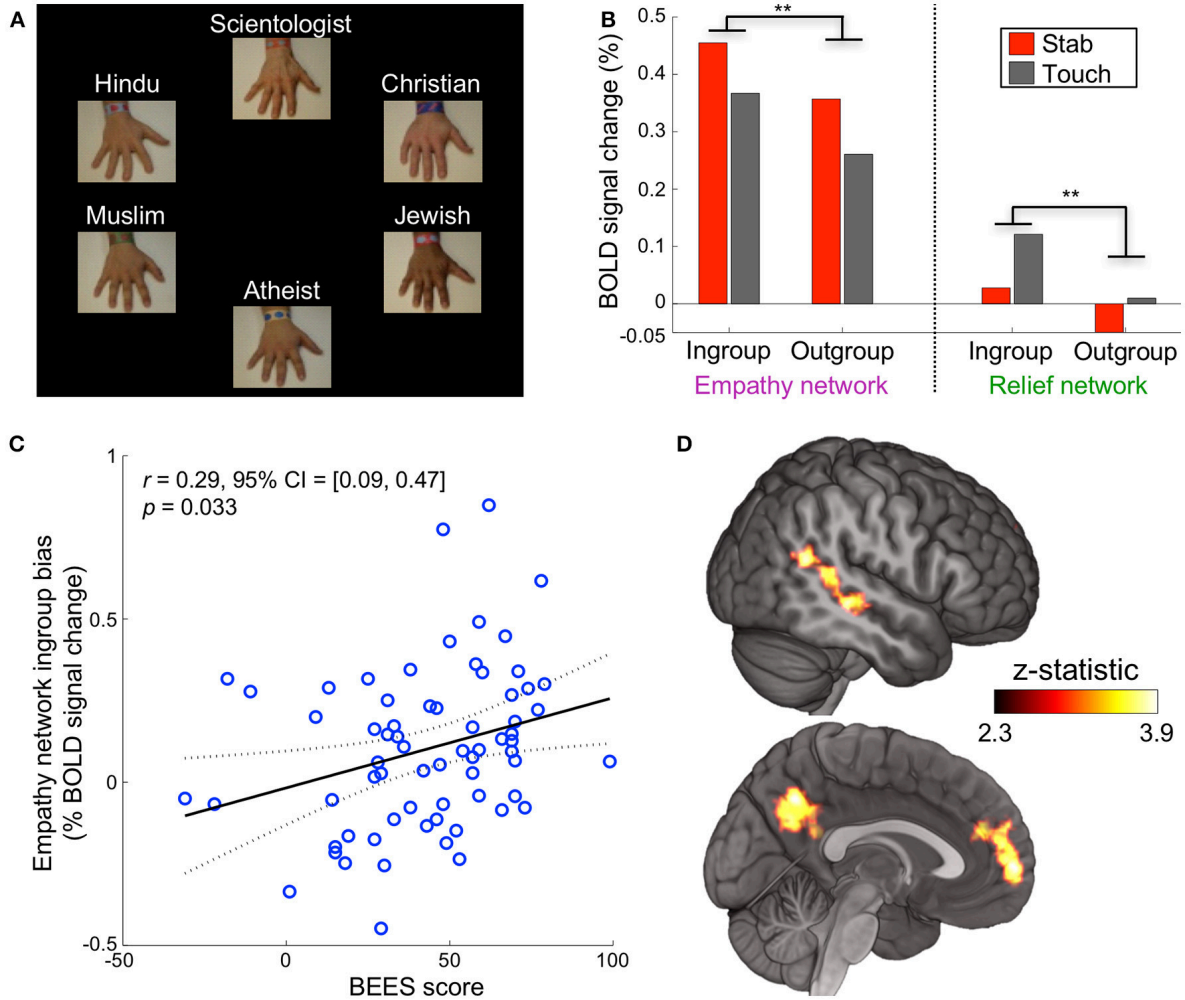
Religious labels modulate empathic neural response. **(A)** Experiment 1 was identical in structure and timing to the baseline block but used religious labels instead of impartial labels. **(B)** When a participant saw their ingroup, in comparison to their outgroup, stabbed or touched, neural activation was significantly higher in the empathy network (^**^p < 0.01 corrected) and relief network (^**^p < 0.01 corrected, repeated measures ANOVA, paired data, *n* = 67 participants). **(C)** Participant scores on the Balanced Emotional Empathy Scale (BEES) correlated with their *ingroup – outgroup* bias in the empathy network. **(D)** A whole-brain *ingroup*>*outgroup* contrast yielded three significant regions: the mPFC, PCC/precuneus, and pSTS/TPJ (*p* < 0.05 FWE). These areas are involved in cognitive empathy and perspective taking; we refer to them collectively as the mentalizing network. The mentalizing network right pSTS is more medial than the relief network right pSTS cluster. No significant voxels appeared in the contrast *outgroup*>*ingroup*.

For the remainder of the experiment, religious group labels were presented above each hand, replacing the previous impartial text labels (Figure 2A). The following 60 trials were identical to the baseline block with the exception of religious hand labeling. For each participant, the religious labels were assigned randomly to the hands, but once assigned, remained with the same hands for the duration.

## Experiment 2: flexibility

**Figure 3:**
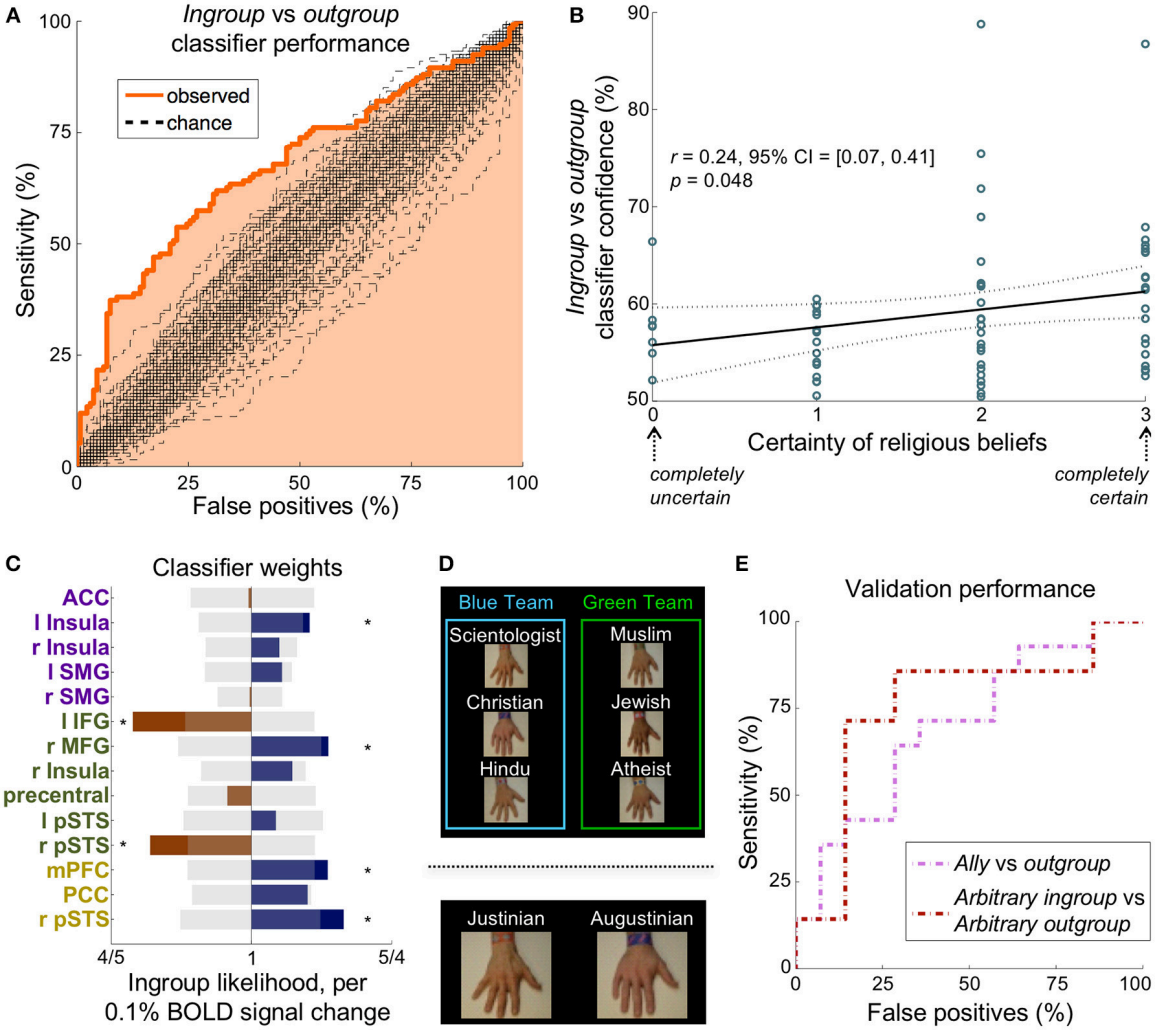
A multivariate classifier discriminates ingroup from outgroup reliably in multiple paradigms. **(A)** The ROC curve for distinguishing the ingroup conditions from the outgroup conditions. The AUC was 68%, which was significantly greater than the chance AUC of 50% (*p* < 0.01, *n* = 268 instances, 100 chance curves shown). **(B)** Participants' self-reported certainty of religious beliefs (scale from 0 to 3) correlated significantly with classifier prediction confidence, suggesting that the strength of an ingroup affiliation may be dependent on certainty. **(C)** The classifier feature weights in the 14 non-visual regions of the empathy (purple), relief (green), and mentalizing (yellow) networks. Translucent gray bars represent the 95% chance interval, and stars demarcate weights that contributed significantly (*p* < 0.05 uncorrected). **(D)** *Top:* Experiment 2 was identical in structure to Experiment 1 except that participants were told that the hands were on two warring teams. The *ally* condition is an outgroup on the same team as the participant's ingroup. *Bottom*: Participants flipped a coin to receive an arbitrary assignment to one of two teams, Justinian or Augustinian, thus defining their *arbitrary ingroup* condition. **(E)** The ROC curves for distinguishing the ingroup condition from the outgroup condition in the two validation paradigms. The classifier determined 64 and 71% of participants' ingroup condition correctly in Experiment 2 (*pink*) and Experiment 3 (*maroon*), respectively.

In Experiment 2, we studied the influence of making a former religious outgroup member more closely connected with an ingroup through an alliance. We assigned the six religions arbitrarily to two groups of three hands: the green team and the blue team (Figure 3D top). A text box said that three of the religions were now at war with the three other religions. The outgroup religions that were on the same team as one's own ingroup religion were considered *allies*.

## Experiment 3: arbitrary teams

In Experiment 3, participants were assigned randomly to one of two fictional groups (the Augustinians and the Justinians) before the fMRI portion of the experiment began. Specifically, participants began by tossing a coin: heads would assign a participant to one team and tails to the other. The assignment relationship was thus randomized across participants, who knew that the assignment was arbitrary. They were next handed a bracelet for their team (either Augustinian or Justinian), which they were instructed to wear. This was intended both to remind them of their team and bond them to it. Aside from the new affiliations, the paradigm was identical to Experiment 1 (Figure 3D bottom).

### Behavioral response

In each experiment, participants were told that the purpose of the study was to examine the effects of pain on memory. They therefore believed they were watching labeled hands being stabbed to see how the presence of pain helped them to remember which hand had been selected on any given trial. To buttress this impression (as well as to quantify alertness), we asked participants on a random 20% of trials to report which religion was associated with the selected hand 10–14 s after the trial. All participants had performance above 80%.

### MR image acquisition

Data were acquired on a Siemens 3T Trio (Erlangen, Germany) scanner. First, high resolution T1-weighted scans were acquired using an MPRage sequence (0.4785 × 0.4785 × 1.0 mm voxels). Functional image acquisition details were as follows: echo-planar imaging, gradient recalled echo; repetition time (TR) = 2,000 ms; echo time (TE) = 40 ms; flip angle = 90°; 64 × 64 matrix, twenty nine 4 mm axial slices, yielding functional 3.4 × 3.4 × 4.0 mm voxels, one ~30 min run.

### Preprocessing

fMRI data processing was carried out using FEAT (FMRI Expert Analysis Tool) Version 6.00, part of FSL 5.0.9 (FMRIB's Software Library, www.fmrib.ox.ac.uk/fsl). The first two volumes from every participant's functional run were discarded. We applied the following pre-statistics processing: motion correction using MCFLIRT (Jenkinson et al., 2002); slice-timing correction using Fourier-space time-series phase-shifting; non-brain removal using BET (Smith, 2002); spatial smoothing using a Gaussian kernel of FWHM 5 mm; grand-mean intensity normalization of the entire 4D dataset by a single multiplicative factor; highpass temporal filtering (Gaussian-weighted least-squares straight line fitting, with sigma = 30 s). All first level analyses and model fitting were conducted in the functional space.

For group level analyses, we registered parameter estimates and contrasts of beta weights to the MNI152 template brain. Registration to high-resolution structural images was carried out using FLIRT (Jenkinson and Smith, 2001; Jenkinson et al., 2002) (full-search, boundary based registration, or BBR). Registration from high resolution structural to standard space was then further refined using FNIRT nonlinear registration (Andersson, 2007a,b) (full-search, 12 DOF, warp resolution 10 mm).

### GLM analysis

We fit a general linear model (GLM) to each participant's time-series data using FSL FILM (FMRIB's improved linear model) with local autocorrelation correction (Woolrich et al., 2001). Six standard motion regressors and individual motion outlier (RMS intensity difference to middle volume, *fsl_motion_outliers*) regressors were added to the model. For each trial condition (*baseline, ingroup, outgroup, arbitrary ingroup, arbitrary outgroup*, and/or *ally*), a set of regressors were included for *stab* and *touch* trials separately, corresponding to the onset of the video of the hand being stabbed or touched. In addition, a regressor for hand selection for each condition was included, corresponding to the time when the particular hand was selected. We also included regressors marking the trial onset across all trials, the times at which questions were asked, and the times at which buttons were selected for the answers. For each regressor, we fit a temporal derivative regressor to allow for slight offsets of peak timings. The durations of each each event were modeled as impulses (0.1 s).

### Group analysis

First, we identified the empathy and relief networks by contrasting the initial 6 *stab* trials with the initial 6 *touch* trials (*baseline stab*—*baseline touch*, Figure 1B). We used FSL FEAT mixed effects modeling (FLAME 1) with outlier deweighting for the group-level contrasts.

Next, we used whole brain search to identify regions outside of the empathy network that responded more when the *ingroup* hand was stabbed painfully. Again, we used FLAME 1 with outlier deweighting for the group-level contrasts *ingroup*-*outgroup*. Contrasts between *ingroups* and *outgroups* were conducted on all participants who had definable *ingroups* and *outgroups* (*n* = 67; agnostics were excluded since they had no *ingroup*). All univariate statistics were corrected for multiple comparisons using Family-wise error (FWE) (Woo et al., 2014).

Note that we chose not to analyze results by the specific religious groups, but instead by looking at *ingroup* and *outgroups* only (irrespective of the religion of the individual participants). Our choice stemmed from the risk of poor or politically-motivated interpretations that could arise erroneously from insufficient statistical power.

### Multivariate classification

We implemented an L^2^-logistic regression classifier to distinguish participants' ingroup condition from their outgroup condition, using BOLD signal change in selected ROIs as predictive features. To rule out a classification based on label text (Petersen et al., 1990; Cohen et al., 2000; McCandliss et al., 2003), we did not use visual areas from the Harvard-Oxford atlas (Frazier et al., 2005; Desikan et al., 2006; Makris et al., 2006) as features.

We trained the classifier on *ingroup* vs. *outgroup* (Experiment 1) using the 11 non-visual ROIs from the empathy and relief networks (derived from our GLM analysis) as predictive features. We did not include the mentalizing network (see Experiment 1 results) because it was derived from the contrast *ingroup-outgroup* and therefore was non-independent from the classification. We assessed performance using leave-one-participant-out cross validation (Figure S10A). We then retrained the classifier on the Experiment 1 data, this time including the empathy, relief, *and* mentalizing network ROIs as features, and applied it to the validation sets: *ally* vs. *outgroup* (Experiment 2) and *arbitrary ingroup* vs. *arbitrary outgroup* (Experiment 3).

We used each stimulus condition (stab and touch) as a separate instance for the classifier, yielding 4 instances per participant in all ingroup vs. outgroup classifications. In the Experiment 1 *ingroup* vs. *outgroup* classification, each participant had the following instances: *ingroup touch, ingroup stab, outgroup touch*, and *outgroup stab* (67 participants × 4 instances = 268 instances). In the Experiment 2 *ally* vs. *outgroup* classification, each participant had the following instances: *ally touch, ally stab, outgroup touch*, and *outgroup stab* (14 participants × 4 instances = 56 instances). In the Experiment 3 *arbitrary ingroup* vs. *arbitrary outgroup* classification, each participant had the following instances: *arbitrary ingroup touch, arbitrary ingroup stab, arbitrary outgroup touch*, and *arbitrary outgroup stab* (14 participants × 4 instances = 56 instances).

We used the standard metric—receiver operator characteristic (ROC) area under the curve (AUC)—as the statistic of interest for measuring the performance of the classifier (Swets, 2014). All classifications were between two classes with equal numbers of instances and thus chance AUC was 50%. To assess the significance of our predictions, we used standard permutation testing to build the null distribution: how well our models might have performed purely by random chance (Good, 2013). In each statistical case, we did the following: we shuffled the outcome across participants so there was no relationship between the potentially predictive features and the condition (Figure S10B). We then conducted the same process of training and validation on these permuted datasets. We repeated this procedure for 20,000 unique permutations to estimate the probability distribution of all our reported summary statistics empirically. Said another way, we built an estimate for how aspects of our model might have turned out, purely by random chance. The *p*-value is the fraction of randomly permuted dataset that resulted in an outcome equal to or more extreme than that observed within the original data.

Each participant's exemplars were brought into a common space, separately, by demeaning their average activation. Specifically, each participant's average activation, (across all the ingroup/outgroup conditions of interest) was subtracted from each condition; thus, greater than 0 signified more activation than their average, and less than 0 signified less activation than their average (Figure S11A left and middle). We ascertained maximum participant-level accuracy by averaging together stab and touch instances in each class for each participant (Figure S11A right), and then applying the classifier weights to those values. For example, in Experiment 1, *ingroup stab* and *ingroup touch* were averaged for each participant to form a single *ingroup* instance; this was done likewise with *outgroup*. In each cross-validation fold, the training model was applied to a participant's individual conditions (*ingroup stab, ingroup touch, outgroup stab*, and *outgroup touch*) to assess AUC and, in parallel, to *ingroup* and *outgroup* to assess accuracy (Figure S11B left). In Experiments 2 and 3, there was no cross-validation and thus the ingroup vs. outgroup model weights were applied, at once, to each of the 14 participant's average ingroup and outgroup conditions (Figure S11B right). The reason for the averaging is that classifier significance is best assessed on the rawest form of the data, whereas averaging improves accuracy by reducing noise.

Averaging each participant's touch and stab trials together for the ingroup and then for the outgroup conditions left only 2 instances per participant: an ingroup and an outgroup condition. Since they were both demeaned, they were the negative of each other by definition, and summed to 0 necessarily. Consequently, there was one unique value only per participant in this classification, which was precisely what we were interested in testing: the classifier's accuracy (right or wrong) in predicting each participant's ingroup and outgroup. As a result, sensitivity was equal to specificity, so the ROC curves were symmetric. We performed all classifications in MATLAB using the LibLinear toolbox (Fan et al., 2008).

### Statistics

Unless otherwise indicated, scalar nonparametric tests (permutation tests and bootstraps) were implemented with 20,000 iterations. Each test type was corrected for multiple comparisons with the Holm-Bonferroni procedure (Holm, 1979): (i) the two (empathy and relief networks) repeated measures ANOVAs (Figure 2B); (ii) the three correlations (BEES vs. empathy network ingroup bias, classifier confidence vs. certainty of religious beliefs, and *baseline stab*—*baseline touch* in the empathy and relief networks) (Figures 2C, 3B, Figure S3B); (iii) the three classifications (*ingroup* vs. *outgroup, ally* vs. *outgroup, arbitrary ingroup* vs. *arbitrary outgroup*) (Figures 3A,E). We list 95% confidence intervals for the mean value of the statistic of interest in square brackets. All correlations calculations are linear (Pearson) and non-Frequentist parameter likelihoods are quantified by Bayes factor (BF).

## Results

### Localizing empathy

Whole brain contrasts for *baseline stab*>*baseline touch* and *baseline touch*>*baseline stab* yielded 6 and 7 significant neural clusters of signal change, respectively (*p* < 0.05 FWE, Figure 1B, Figure S2, Table S1). Consistent with previous findings, we interpret the regions localized by *baseline stab*>*baseline touch* as the empathy-for-pain network (henceforth simply the *empathy* network); it contains both affective (insula / anterior cingulate) and sensorimotor (lateral occipital, fusiform, supramarginal gyrus) components (Decety, 2010; Hein et al., 2010; Lamm et al., 2011; Zaki et al., 2012). The network we identified with *baseline touch*>*baseline stab* has not been reported previously. Within the context of the experiment, one interpretation is that the touch translates to relief that the hand was not stabbed; we therefore provisionally refer to this as the relief network. This network comprised the left inferior frontal gyrus, right middle frontal gyrus, right posterior insula, precentral gyrus, precuneus, bilateral posterior superior temporal sulci (pSTS), and bilateral angular gyri. Several of the regions in both networks have been implicated in neural resonance experiments (Iacoboni et al., 1999), and shared representation paradigms (Lawrence et al., 2006; Lamm et al., 2007). Across participants, the responsiveness of these two networks was linearly correlated (*r* = 0.46, *p* < 10^−4^ corrected, Figure S3), possibly because the amount of relief one experiences when a stab is avoided is related to how much empathy one has when watching a stabbing.

### Experiment 1: are one's neural responses modulated by the religion of another?

After the baseline block, the text label of each hand (e.g., “Hand #1”) was replaced with one of six religious affiliations (*Christian, Muslim, Hindu, Jewish, Scientologist*, or *atheist*) for the duration of the experiment (Figure 2A). A hand labeled with a participant's self-reported religion is referred to as the *ingroup* condition, while the other religious beliefs comprise the *outgroup* condition (Figure S1). Neural activation for ingroups was significantly higher than for outgroups in the empathy and relief networks when a participant saw the hands stabbed or touched (*p* < 0.01 corrected for each, repeated measures ANOVA, paired data, *n* = 67 participants, Figure 2B, Figures S4, S5). We refer to this activation difference (averaged across stab and touch conditions) as the “ingroup bias.”

Given that activation in empathy-associated regions has been shown to correlate with psychometric measures and behavioral outcomes (Singer, 2004; Singer et al., 2006), we investigated whether the ingroup bias might correlate similarly with self-reported empathy. Participants' ingroup bias in the empathy network were positively correlated with their scores on the Balanced Emotional Empathy Scale (BEES) (Mehrabian, 1997) (*r* = 0.29 [0.09, 0.47], *p* = 0.03 corrected, Figure 2C). This bias likely is driven by a positive correlation of BEES (BF = 7.5 *substantial*) with the ingroup response and a negative correlation with the outgroup response (BF = 21 *very strong*, Figure S6).

A whole brain contrast for *ingroup*>*outgroup* (each combining stab and touch conditions) yielded three ROIs: the medial prefrontal cortex (mPFC), posterior cingulate cortex (PCC)/precuneus, and right posterior superior temporal sulcus/temporoparietal junction (pSTS/TPJ) (Figure 2D). These areas are involved in cognitive empathy (also known as perspective-taking, theory of mind, or mentalizing) (Preckel et al., 2018); we refer to them collectively as the *mentalizing* network (Mitchell et al., 2005; Zaki et al., 2012). There were no significant voxels in the contrast *outgroup*>*ingroup*. The empathy and mentalizing networks we localized are highly consistent with previous findings (Zaki et al., 2012) and their interplay has been well documented (Hooker et al., 2008; Schnell et al., 2011; Christov-Moore et al., 2017) (Figure S7). Group distinctions, therefore, may rely on mental simulation that is more involved for ingroup members than for outgroup members.

### Does activity distinguish religious ingroups from outgroups?

We used average activation in each of the non-visual regions (Figure S8) of the empathy and relief networks (Figure 1B) in a logistic regression to distinguish *ingroup* from *outgroup*. A univariate model, using the average activation of the empathy network, discriminated the *ingroup* conditions (stab and touch) from the *outgroup* conditions (stab and touch) with an accuracy of only 60%. A multivariate model, however, discriminated the *ingroup* conditions (stab and touch) from the *outgroup* conditions (stab and touch) with a receiver operator characteristic (ROC) area under the curve (AUC) of 68%, which was significantly different from chance (*p* < 0.01, *n* = 268 instances, Figure 3A). This model distinguished *ingroup* from *outgroup* correctly for 72% of participants (*n* = 67). Including all other non-visual brain regions as features yielded similar results (AUC = 69%, participant accuracy = 70%, Figure S9). Removing empathy-associated regions from this expanded classification, however, decreased discriminability (AUC = 57%, *p* = 0.21). Although specific to this classifier, these results putatively demonstrate that the empathy, relief, and mentalizing networks may be both sufficient and necessary to distinguish *ingroup* from *outgroup*.

Interestingly, the classifier in Figure 3A correctly distinguished the *ingroup* and *outgroup* conditions in all participants who self-identified as atheist, suggesting the bias is not so much about religion as about affiliation. Participants' self-reported certainty of their belief (on a scale from 0 to 3) correlated significantly with classifier prediction confidence (*r* = 0.25 [0.07, 0.41], *p* = 0.048 corrected, Figure 3B). In other words, a person's certainty in their group's principles relates to the ease of classifying their ingroup from neural data.

### Does our religious-ingroup classification model generalize?

To test the validity and generality of our classifier (ingroup vs. outgroup), we conducted two validation experiments using modified versions of Experiment 1 and 28 independent participants. We retrained the classifier on the Experiment 1 data, this time additionally including the mentalizing network ROIs as features (the validation classifications were independent of the derivation of the mentalizing network) (Figure 3C).

At the beginning of Experiment 2, hand labels were distributed evenly and randomly between two “teams” that were said to be at war (Figure 3D *top*). The two outgroups on the same team as the ingroup were defined as the *ally* group (Figure S1). Our classifier discriminated the *ally* conditions from *outgroup* conditions with an AUC of 65% (*p* < 0.05 corrected, *n* = 56 instances), corresponding to accurate condition identification in 64% of participants (*n* = 14, Figure 3E *pink*).

In Experiment 3, participants were assigned randomly (by a coin flip by the participant) to the Augustinian or Justinian team. They were then given a bracelet with their team name, and informed that the Augustinians and Justinians were two warring tribes. Hands were labeled as Augustinian or Justinian (Figure 3D *bottom*). The hand labeled with the participant's own team defined the *arbitrary ingroup* condition, while the opposing team's hand defined the *arbitrary outgroup* condition (Figure S1). Our classifier discriminated the *arbitrary ingroup* conditions from *arbitrary outgroup* conditions with an AUC of 70%, which was significantly different from chance (*p* < 0.05 corrected, *n* = 56 instances), corresponding to the accurate condition identification in 71% of participants (Figure 3E *maroon*).

## Discussion

The ingroup bias (difference between ingroup and outgroup empathic response) was elicited by the simple difference in a single-word text label on a hand, without any interpersonal interaction or additional information. These findings are consistent with the behavioral results of minimal group theory: that ingroup/outgroup discrimination occurs in the presence of even minimally-differentiating information (Tafjel and Turner, 1979). Additionally, our results provide spatial localization to an effect demonstrated in a recent EEG paper, which found an event-related potential (ERP) difference in the frontal lobe between religious ingroups and outgroups, using only Christians and atheist participants (Huang and Han, 2014).

Our correlational data suggest this bias stems from an increase in neural response for ingroup stimuli, and a decrease in response for outgroup stimuli. While initially counterintuitive, this result—that participants who consider themselves more empathic show a larger ingroup bias—might be explained by ambiguity in the BEES' hypotheticals. In questions, such as “*it would be extremely painful for me to have to convey very bad news to another*,” the BEES test does not define who the other person is. When answering empathy-related questions, participants may not imagine a nondescript person, but instead, by default, a member of their ingroup. Thus, it may not be surprising to find a positive relationship between self-reported empathy for one's own ingroup and a neural correlate of that bias.

The results of experiments 2 and 3 suggest that an ingroup bias can be extended or generated arbitrarily. In Experiment 2, neural activation to outgroup religions on the ingroup member's team was more like activation in response to the ingroup. Experiment 3 demonstrates that group distinctions can be manufactured arbitrarily, as neural differences were present after a visibly random group assignment. The behavioral implications of these results are consistent with findings that ingroup distinctions can be modified flexibly and created arbitrarily on the basis of eye-color (Byrnes and Kiger, 1990), assigned role (Haney et al., 1972), mutual experience (Sherif, 1961), and perceived similarity (Ruckmann et al., 2015).

Our results shed light on a recent finding that responses typically thought of as empathic in nature, maybe instead be attributable to a sense of body ownership (Bucchioni et al., 2016). Note that the hands in our present experiment were displayed upside-down (a third-person perspective), yet we still observed a response in well-established empathy-related regions. While our results do not rule out a role for ownership in response, they preclude ownership-dependent modulation that often accompanies a first-person perspective.

Human allegiances often are more complex than a binary classification between ingroups and outgroups. Nonetheless, empathy regions allow for the classification of long-held, newly-modified and arbitrarily-formed ingroups and outgroups. This is the first report of a single machine learning model on neural activation that generalizes to multiple representations of ingroup and outgroup. Our multivariate analysis performed similarly on atheist participants and generalized to flexible and arbitrary teams, suggesting that our classifier is not specific to religion. Instead, we interpret our findings as evidence of brain activity differences based on group affiliation. We did not have sufficient data to make a statistically-significant inference regarding the degree to which participants perceived other religions as more or less related to their own (e.g., would a Christian participant respond more empathically to a Jewish-labeled hand than an atheist-labeled hand?).

Using a single, group-level machine learning model—rather than individually-specific models—to predict ingroup/outgroup affiliations might have reduced our classification accuracy by ignoring the nuances of each participant's spatio-functional brain organization. However, our model offers distinct advantages in both interpretability and applicability. Our model can be applied immediately to additional participants and similar paradigms without first needing to acquire data with which to train the parameters of a participant-specific classifier. Although it is tempting to interpret the biological meaning of brain regions found to be significant features in our multivariate model (Figure 2C), we do not; Haufe and colleagues have demonstrated clearly that, in most cases, classifier weights cannot be interpreted individually (Haufe et al., 2014). The utility from the classification portion of this experiment lies in predictions only.

Bolstered by recent TMS results suggesting a causal link between mentalizing regions, religious beliefs, and empathic behavior (Holbrook et al., 2016; Christov-Moore et al., 2017), our present paradigm and classifier may prove useful as an objective diagnostic tool to measure the magnitude of one's ingroup biases (e.g., political party, gender, race). It might therefore prove useful for measuring the efficacy of different interventional programs to reduce the bias between ingroup and outgroup.

## Author contributions

DV and DE conceived the experiments. DV constructed the stimuli and scanned all participants. RS and DV analyzed data under the supervision of DE and MC. MC directed all classification efforts. RS, DV, MC, and DE wrote the paper. DV and RS contributed equally and are co-first authors.

### Conflict of interest statement

The authors declare that the research was conducted in the absence of any commercial or financial relationships that could be construed as a potential conflict of interest.
