# Supplementary material for: Empathic Neural Responses Predict Group Allegiance
Source: Front Hum Neurosci. 2018 Jul 31;12:302. doi: 10.3389/fnhum.2018.00302 (PMC6079240; doi:10.3389/fnhum.2018.00302)
Supplement: Supplementary file 3 [file Data_Sheet_1.pdf]

Empathic neural responses predict group allegiance - Supplement

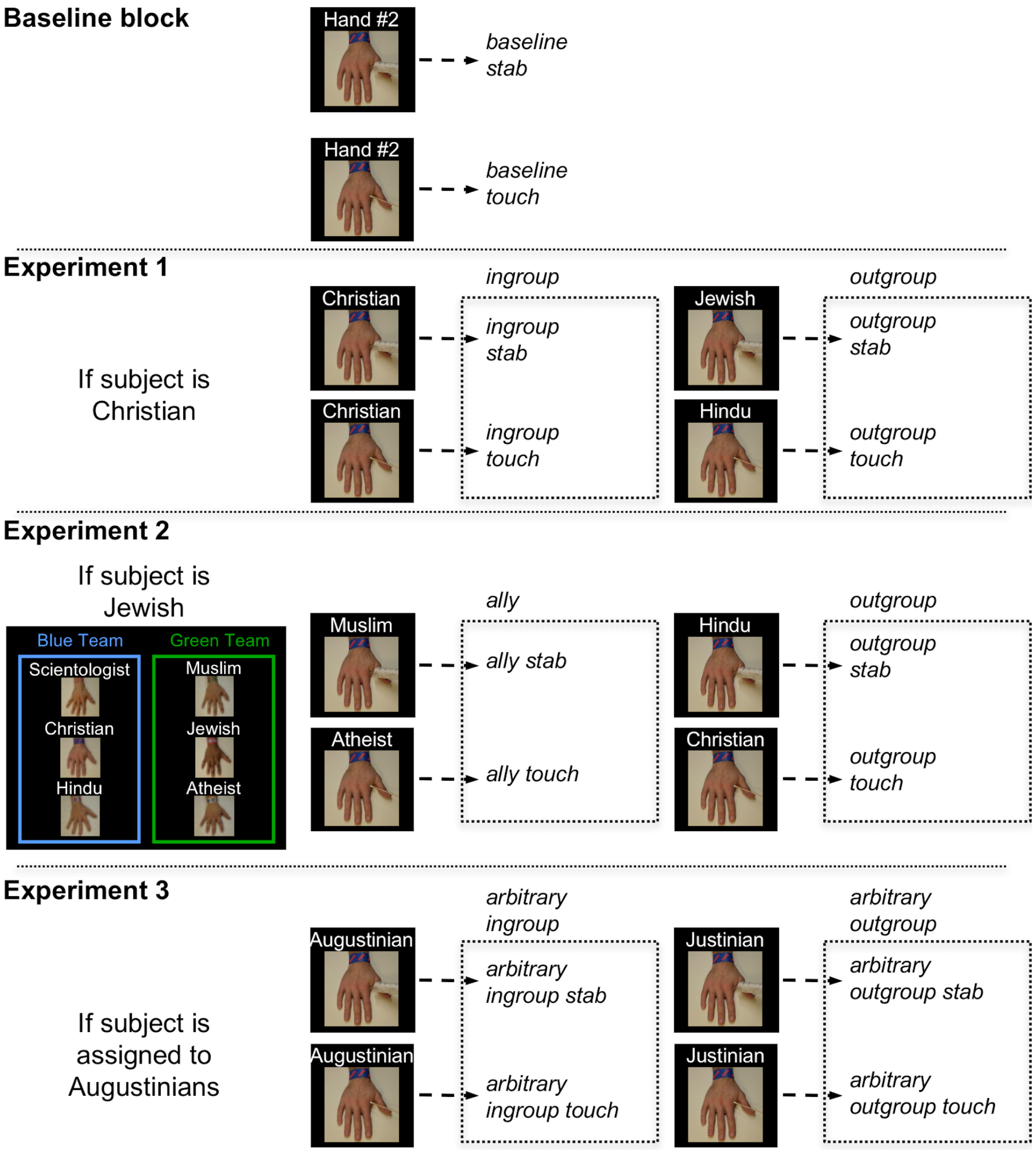

Fig. S1. Examples of the correspondence between paradigm stimuli and condition names. In Experiment 1, the outgroup condition religions are representative examples; the actual condition would include any religious affiliations other than the participant's self-reported religious affiliation. Similarly, in

Experiment 2, the participant's self-reported religious affiliation was Jewish, and thus stabs and touches to the hands labeled Muslim and Atheist would comprise the *ally* conditions, whereas stabs and touches to the hands labeled Scientologist, Christian, and Hindu would constitute the *outgroup* conditions.

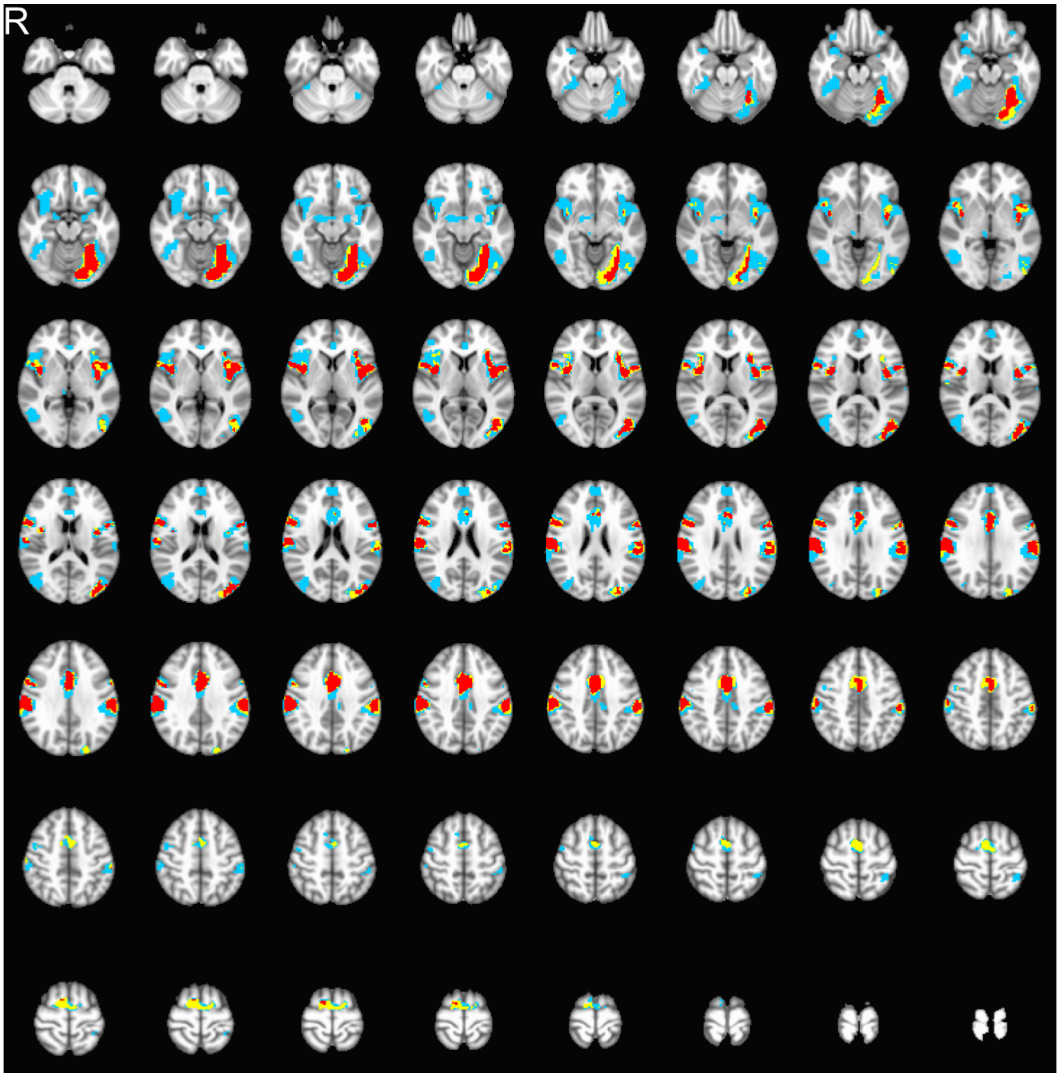

Fig. S2. We localized the empathy network using multiple methodologies. Ordinary Least Squares was coupled with Statistical Parametric Mapping (SPM), FLAME, or permutations testing with FSL *randomise.* All methods identified a similar empathy network for the contrast *baseline stab>baseline touch*. **Red** indicates voxels found to be significant for all three approaches, **yellow** indicates significance for two approaches, and **blue** indicates significance for one approach only. Most blue regions were significant only with SPM, which may be due to a slightly larger smoothing kernel (8mm vs 5mm). Images are radiologic convention. We used the FLAME results exclusively in our analyses.

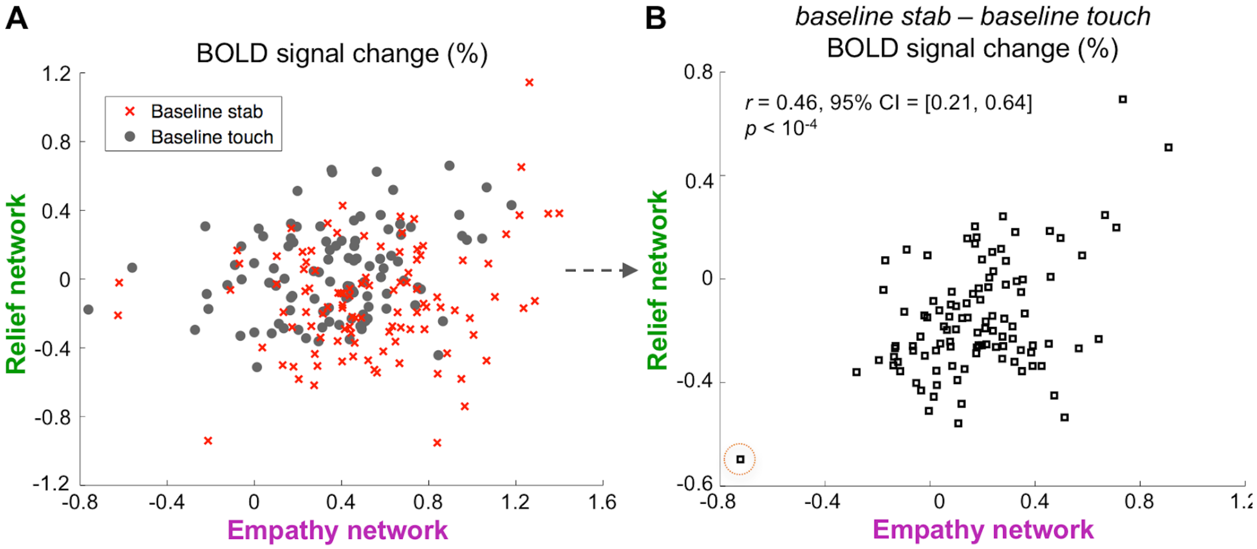

Fig. S3. Stabs and touches. A. *Baseline stab* and *baseline touch* activation in the empathy and relief networks. Each marker is a single participant's condition. **B.** Activation for the contrast *baseline stab* - *baseline touch* was correlated positively between the empathy network and relief network; participants who showed larger activation in the empathy network were likely to show more activation in the relief network (r = 0.46, $p < 10^{-4}$ corrected). Each marker is a participant. After removing the one potential outlier (marked with a dotted orange circle), this correlation remains highly significant.

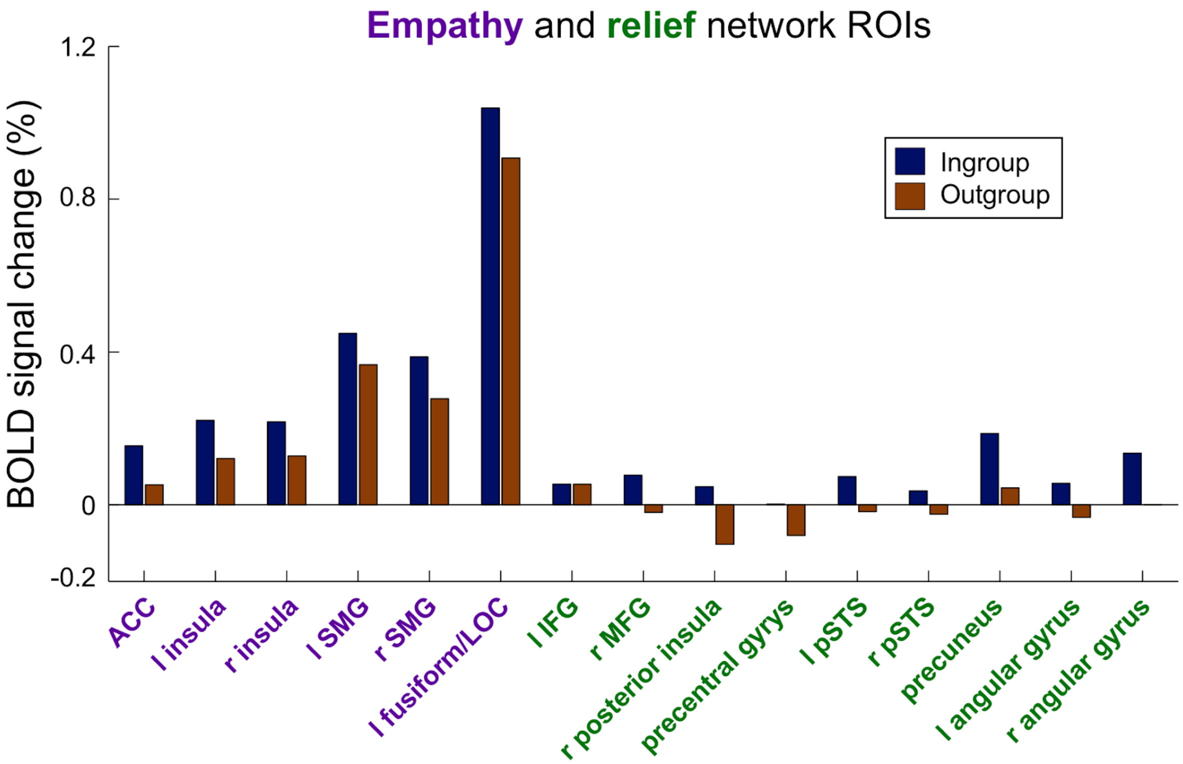

Fig. S4. Religious ingroup bias in the empathy (purple) and relief (green) networks. A. Participants

showed, on average, larger activation in response to their *ingroup* than their *outgroup* within most ROIs of the empathy and relief networks. Thus, the average network ingroup activation bias (**Fig. 2B**) does not appear to be driven by a few individual ROIs, but rather, is present in most regions. Data are paired. *Key***:** ACC = anterior cingulate cortex, SMG = supramarginal gyrus, LOC = lateral occipital cortex, IFG = inferior frontal gyrus, MFG = middle frontal gyrus, pSTS = posterior superior temporal sulcus.

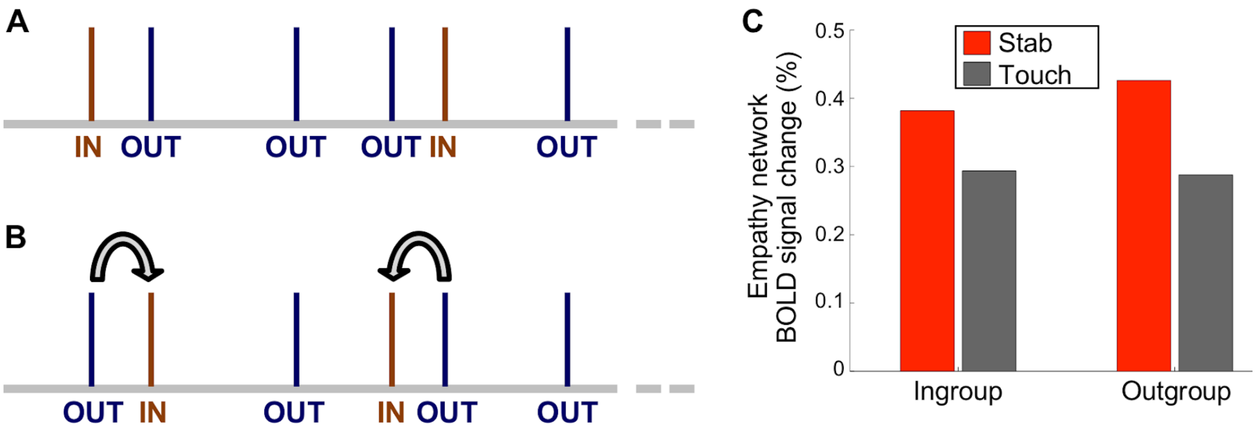

Fig. S5. The *ingroup* – *outgroup* bias cannot be attributed to differences in the number of trials. To account for frequency effects (there were more *outgroup* than *ingroup* trials), we ran a control analysis in which *ingroup* trial labels were swapped with the closest matching *outgroup* trial labels. The results showed no significant *ingroup* effect, indicating that the main results were not due to an imbalanced number of trials. **A.** The stick diagram represents a schematic of the true timing of *ingroup* (IN) and *outgroup* (OUT) trials. Condition type (stab or touch) labels are not shown here for simplicity. **B.** Trials constituting the *ingroup* condition were swapped with the *outgroup* trials that were closest in time (stimulus type—*stab* or *touch*—was conserved). **C.** With the *ingroup* trials swapped, the group effect of the empathy network was no longer observed (*stab*: p = 0.59, *touch*: p = 0.92; percentile permutations test).

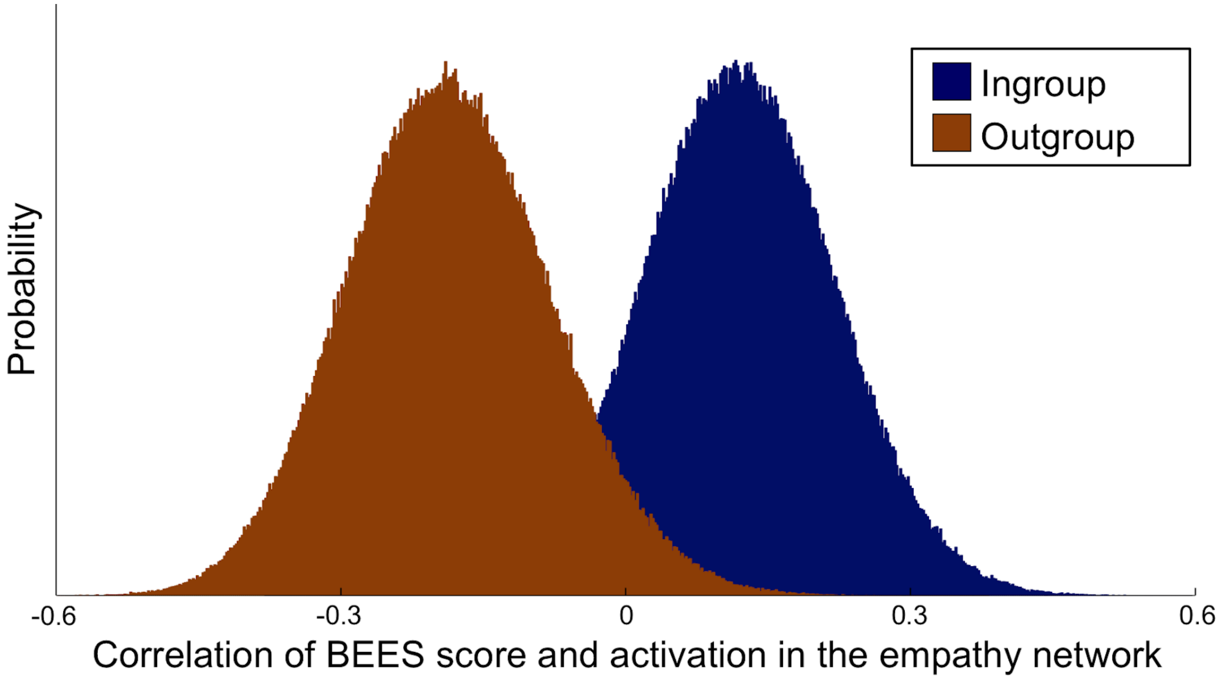

Fig. S6. With self-reported empathy, *ingroup* correlates positively while *outgroup* correlates negatively. We used 500,000 bootstraps to generate a distribution of the Pearson correlation of each participant's empathy network activation and BEES score for both the ingroup and outgroup conditions, analogous to a Bayesian inference with no prior. 88% of the ingroup bootstraps were greater than 0 and thus the Bayes factor for a positive correlation relative to a negative correlation of 7.5. 95% of the outgroup bootstraps were greater than 0 and thus the Bayes factor for a positive correlation relative to a negative correlation of 21. The strength of evidence supporting the ingroup condition correlating positively with BEES and the outgroup condition correlating negatively with BEES is substantial and very strong, respectively(Jeffreys, 1961).

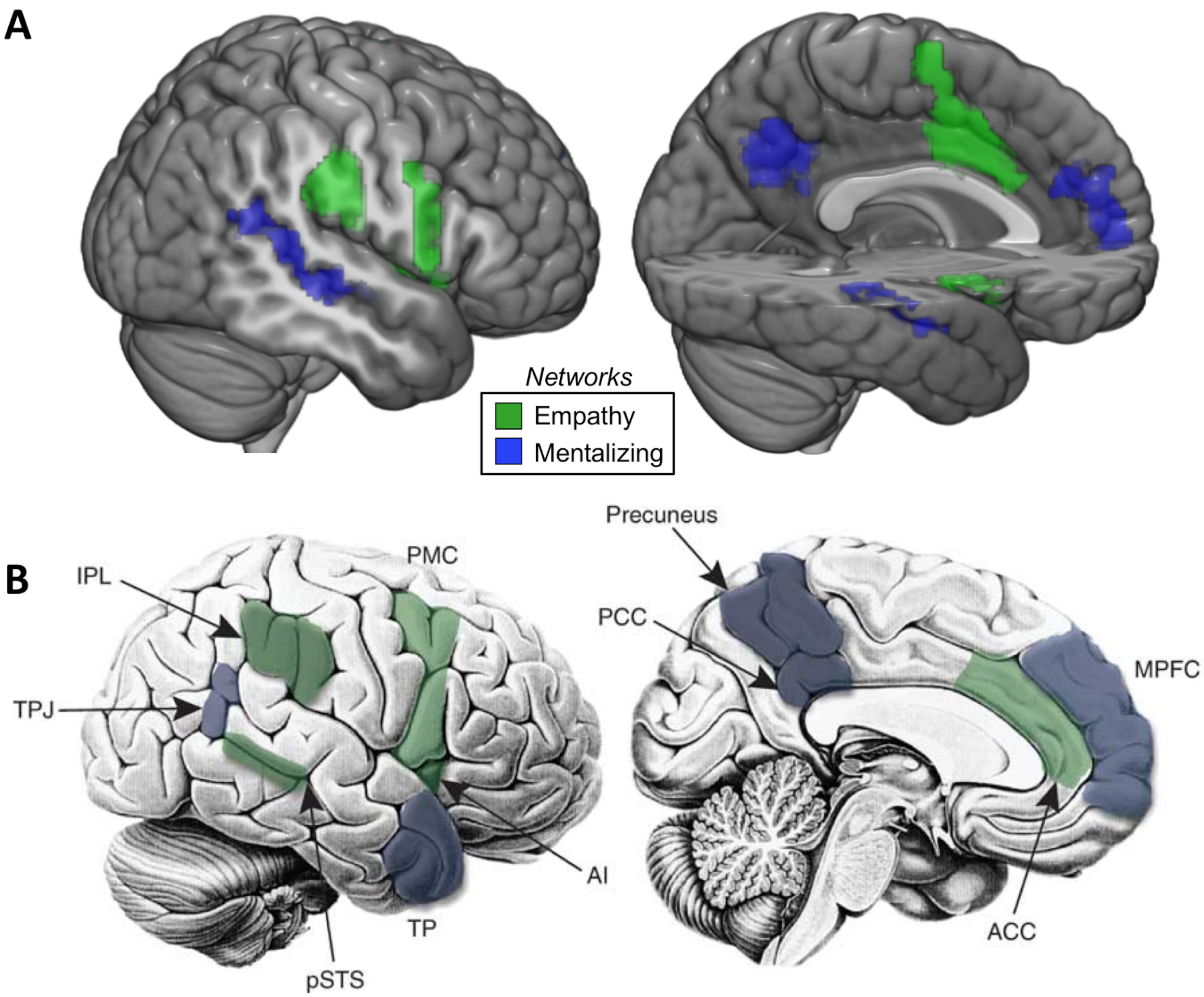

Fig. S7. Our empathy and mentalizing networks are consistent with previous findings. A. The empathy and mentalizing networks localized in our univariate analysis. **B.** *Affective empathy* and *mentalizing* networks reproduced from a 2012 *Nature Neuroscience* review article on empathy(Zaki and Ochsner, 2012). *Key*: ACC = anterior cingulate cortex, AI = anterior insula, IPL = inferior parietal lobule, MPFC = medial prefrontal cortex, PMC = premotor cortex, pSTS = posterior superior temporal sulcus, TP = temporal pole, TPJ = temporal-parietal junction. Notably, our *ingroup* - *outgroup* contrast shows bilateral temporal pole activation just below the $p < 0.05$ FWE significance threshold.

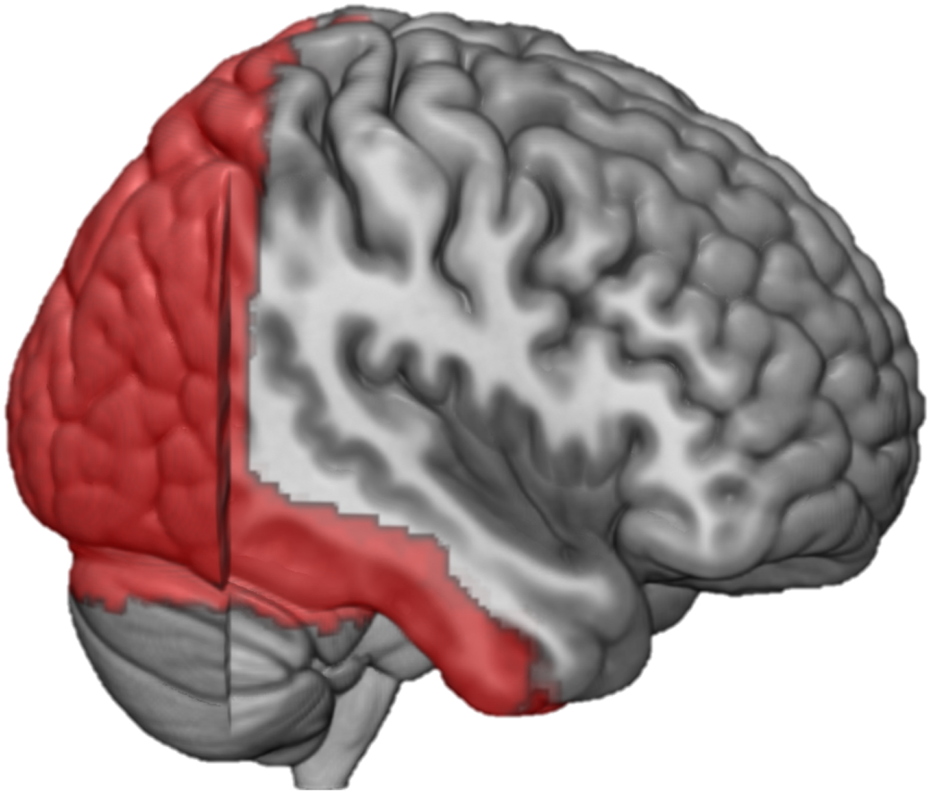

Fig. S8. Visual exclusion mask. Areas in the Harvard-Oxford atlas considered to be significantly visual and thus not included in any classification analyses.

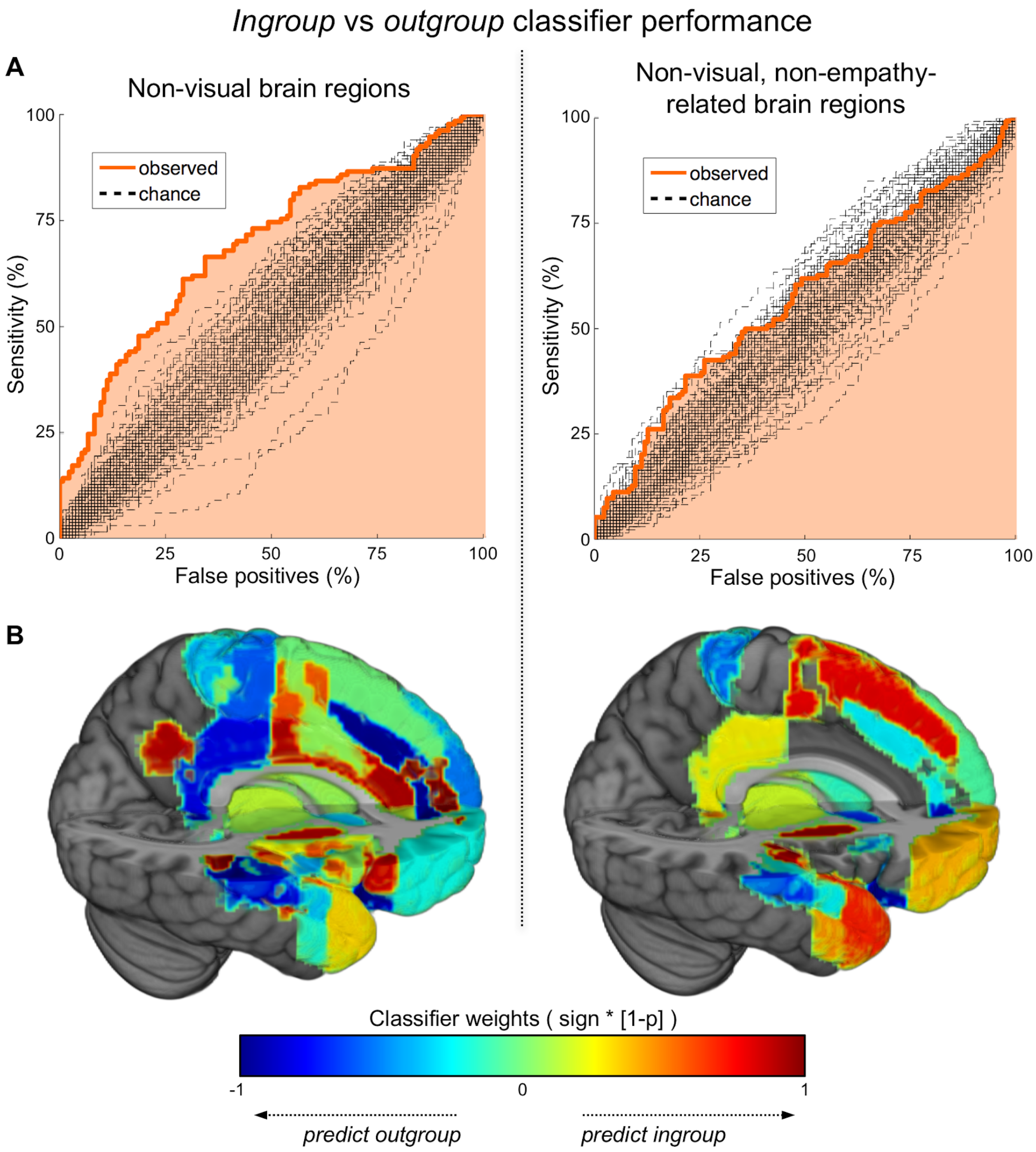

Fig. S9. Whole brain ingroup vs outgroup classifier performance. **A.** ROC curves for a classifier discriminating *ingroup* vs *outgroup* using non-visual areas of the empathy, relief, and mentalizing networks as well as the Harvard-Oxford parcellation (AUC = 69%, $p < 10^{-3}$, *left*), or using non-visual areas of the Harvard-Oxford atlas that do not overlap significantly with the empathy, relief, and mentalizing networks (AUC = 57%, $p = 0.21$, *right*). 100 chance curves shown in each plot. **B.** Weights for the two classifiers. Positive (red) regions predict ingroup and negative (blue) regions predict outgroup; magnitude

is 1 - p; thus larger magnitude reflects weights differ more from the null permutation distribution. p-values are uncorrected.

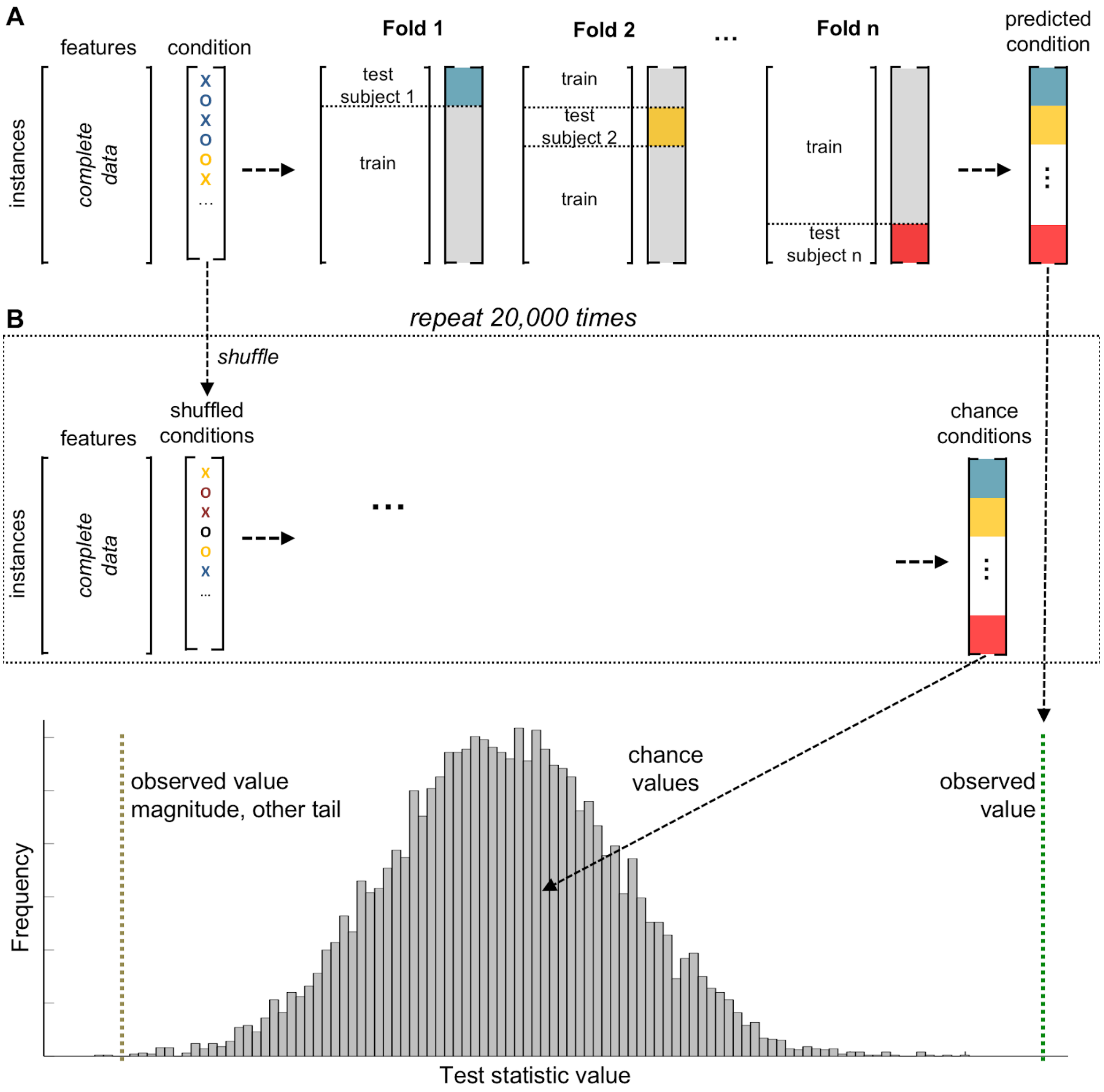

Fig. S10. Statistical methods. **A.** In each fold of our cyclical leave-one-participant-out cross-validation paradigm, a classifier was trained on the features and labels from all-but-one participant's conditions, and then tested on the 1 excluded participant's features to make a prediction for each instance. Each instance was included in exactly one fold and thus had a single prediction. Each predicted condition was compared with the actual condition label to assess the model's performance. **B.** To simulate chance classifier performance, we used permutation testing: randomly shuffling outcomes to break any link between the

predictive features and the condition. After 20,000 repetitions we compared our actual classifier performance to performance in the shuffled cases to calculate an empirical p-value: the fraction of the chance distribution more extreme than the observed test statistic.

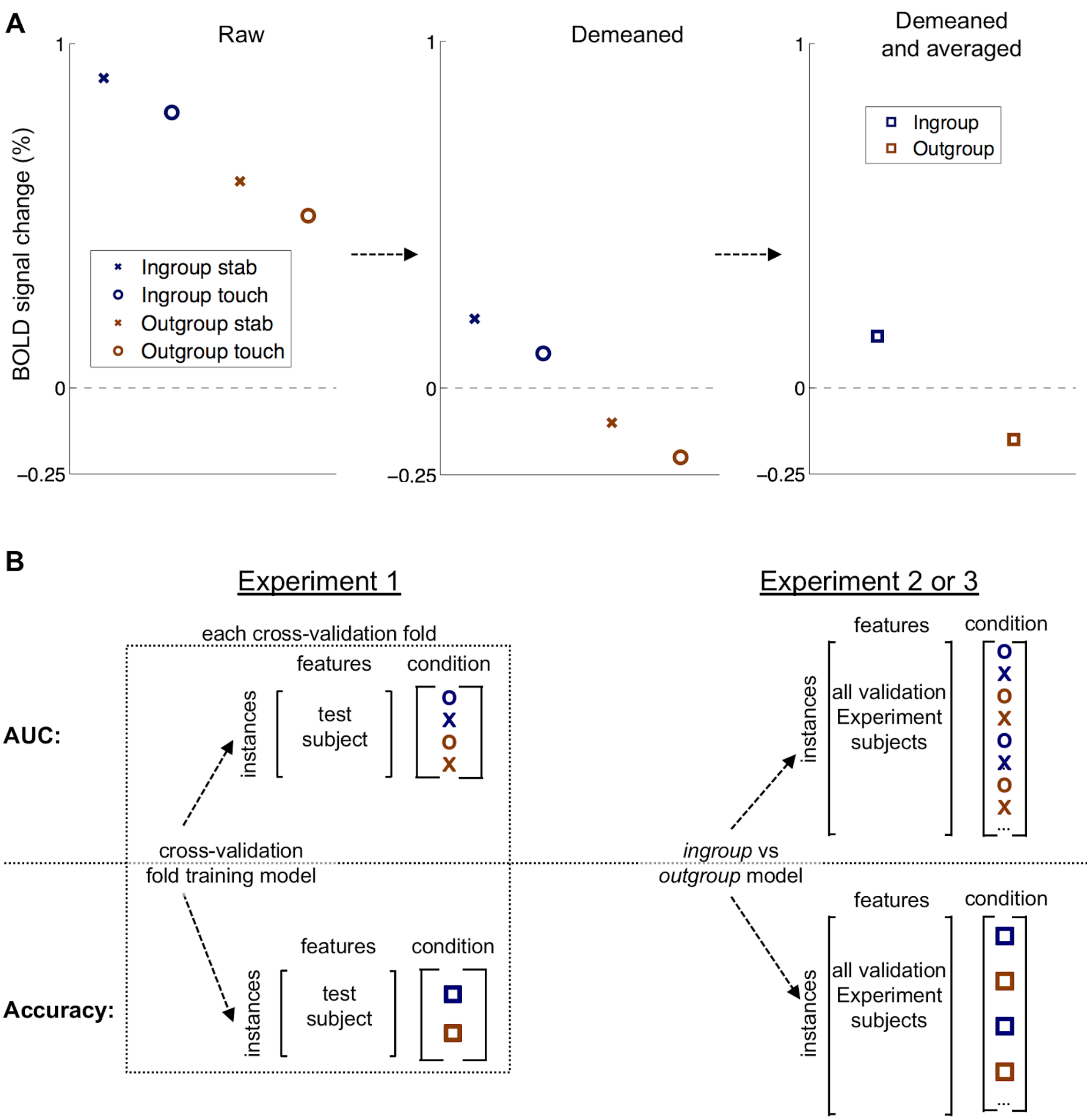

Fig. S11. Instance extraction and classification use. A. Each participant's exemplars were brought into a common space separately by demeaning each participant's average activation over the conditions of interest. Additionally, we averaged the ingroup and outgroup conditions across stimulus type for use in assessing maximum participant-level accuracy. **B.** We ascertained condition-level AUC by using all

parameter estimate images; we ascertained maximum participant-level accuracy by averaging together stab and touch instances in each class, for each participant, and then applying the classifier weights to those values.

	# voxels	*p* value	Z-statistic center of gravity (mm, MNI space)		
			**x**	**y**	**z**
**Empathy network**					
Left Fusiform Gyrus & Lateral Occipital Cortex	2899	1.8E-11	-27.6	-74.0	-5.1
Anterior Cingulate Cortex	1661	1.2E-07	1.0	4.9	46.8
Left Anterior Insula	1100	1.8E-05	-43.6	4.9	7.6
Right Anterior Insula	1005	4.3E-05	49.2	6.9	12.9
Right Inferior Parietal Lobule	966	6.2E-05	60.6	-21.5	31.4
Left Inferior Parietal Lobule	855	1.8E-04	-58.9	-25.7	32.9
**Relief network**					
Left Angular Gyrus & Left Superior Temporal Gyrus, posterior division	2563	1.7E-10	-51.3	-47.5	19.4
- Left Angular Gyrus	1220	6.3E-06	-44.4	-56.6	38.5
- Left Superior Temporal Gyrus, posterior division	1343	2.1E-06	-57.0	-38.4	-0.2
Left Inferior Frontal Gyrus	1507	4.8E-07	-41.2	30.2	22.7
Precentral Gyrus	971	5.9E-05	2.4	-29.4	62.3
Right Superior Temporal Gyrus, posterior division	869	1.6E-04	55.3	-30.4	4.8
Right Angular Gyrus	724	7.1E-04	45.5	-57.5	39.4
Precuneous Cortex	592	3.0E-03	-0.5	-65.9	43.8
Right Inferior Frontal Gyrus	312	7.2E-02	30.3	20.5	53.3
Right Posterior Insula	152	8.3E-01	38.7	-17.3	16.9
**Mentalizing network**					
Medial Prefrontal Cortex	832	2.0E-04	2.1	54.6	12.3
Precuneous / Posterior Cingulate Cortex	818	2.4E-04	1.3	-56.3	34.1
Right Superior Temporal Sulcus, posterior division & Temporoparietal junction	698	8.3E-04	54.1	-34.1	5.2

Supplementary Table T1. Univariate network statistics. Size, significance, and location information for networks we localized using univariate GLM contrasts.

Supplementary Mov. M1. Stimulus paradigm for a representative experiment. The first trial shows an example of a baseline pain trial in which a hand is chosen randomly and stabbed painfully with a needle. Similarly, the next trial depicts a baseline no pain trial, in which a hand is chosen randomly and touched

non-painfully with a cotton swab. In the last three trials depicted, the six hands are each labeled with a religious belief, along with the strength of the conviction of that individual's religion (1-3, 1 being the lowest conviction). The observer identified with one of those religious groups, and that religion would serve as that participant's ingroup.

References

Jeffreys, H. (1961). Theory of probability (3rd edt.) oxford university press. *MR0187257*.

Zaki, J., and Ochsner, K. N. (2012). The neuroscience of empathy: progress, pitfalls and promise. *Nat. Neurosci.* 15, 675–680.
